# Genomic profiling of NSCLC tumors with the TruSight oncology 500 assay provides broad coverage of clinically actionable genomic alterations and detection of known and novel associations between genomic alterations, TMB, and PD-L1

**DOI:** 10.3389/fonc.2024.1473327

**Published:** 2024-11-27

**Authors:** Zachary D. Wallen, Mary K. Nesline, Marni Tierno, Alison Roos, Erica Schnettler, Hatim Husain, Pratheesh Sathyan, Brian Caveney, Marcia Eisenberg, Eric A. Severson, Shakti H. Ramkissoon

**Affiliations:** ^1^ Labcorp Oncology, Durham, NC, United States; ^2^ Illumina, San Diego, CA, United States; ^3^ Moores Cancer Center at UC San Diego Health, La Jolla, CA, United States; ^4^ Labcorp, Burlington, NC, United States; ^5^ Wake Forest Comprehensive Cancer Center, Wake Forest School of Medicine, Department of Pathology, Winston-Salem, NC, United States

**Keywords:** non-small cell lung cancer, genomic profiling, immune checkpoint inhibitors, TMB, PD-L1, clinical utility, targeted therapy, genomics

## Abstract

**Introduction:**

Matching patients to an effective targeted therapy or immunotherapy is a challenge for advanced and metastatic non-small cell lung cancer (NSCLC), especially when relying on assays that test one marker at a time. Unlike traditional single marker tests, comprehensive genomic profiling (CGP) can simultaneously assess NSCLC tumors for hundreds of genomic biomarkers and markers for immunotherapy response, leading to quicker and more precise matches to therapeutics.

**Methods:**

In this study, we performed CGP on 7,606 patients with advanced or metastatic NSCLC using the Illumina TruSight Oncology 500 (TSO 500) CGP assay to show its coverage and utility in detecting known and novel features of NSCLC.

**Results:**

Testing revealed distinct genomic profiles of lung adenocarcinoma and squamous cell carcinomas and detected variants with a current targeted therapy or clinical trial in >72% of patient tumors. Known associations between genomic alterations and immunotherapy markers were observed including significantly lower TMB levels in tumors with therapy-associated alterations and significantly higher PD-L1 levels in tumors with *ALK*, *MET*, *BRAF*, or *ROS1* driver mutations. Co-occurrence analysis followed by network analysis with gene module detection revealed known and novel co-occurrences between genomic alterations. Further, certain modules of genes with co-occurring genomic alterations had dose-dependent relationships with histology and increasing or decreasing levels of PD-L1 and TMB, suggesting a complex relationship between PD-L1, TMB, and genomic alterations in these gene modules.

**Discussion:**

This study is the largest clinical study to date utilizing the TSO 500. It provides an opportunity to further characterize the landscape of NSCLC using this newer technology and show its clinical utility in detecting known and novel facets of NSCLC to inform treatment decision-making.

## 1 Introduction

Lung cancer is the leading cause of cancer-related death, with an estimated 1.8 million deaths worldwide (1). Non-small lung cancer (NSCLC) is the most common type of lung cancer and is comprised of three major histologic subtypes, including adenocarcinoma, squamous-cell carcinoma, and large-cell carcinoma in 40-50%, 20-30%, and 5-10% of diagnoses, respectively (2, 3). Although smoking is a risk factor for all types of lung cancer, squamous-cell carcinoma is strongly associated with smoking (4). Comparative sequencing studies have demonstrated distinct genomic profiles within NSCLC subtypes, with non-squamous NSCLC frequently harboring more alterations in oncogenes, including *KRAS, EGFR, BRAF*, and *MET*, while squamous cell carcinomas frequently have *TP53* and *CDKN2A* mutations (5–8). Oncogenic-driven NSCLCs are typically devoid of other drivers and have distinct patterns of tumor mutational burden (TMB) and PD-L1 positivity (9, 10).

The clinical application of precision medicine has transformed the management of patients with NSCLC, especially for patients with non-squamous NSCLC (11). The identification of oncogenic drivers in NSCLC, some of which are therapy-associated targets, has enabled a shift from chemotherapy to genomics-informed targeted therapy (5, 9, 12). Systemic therapy for patients with advanced and metastatic NSCLC is currently best tailored according to the presence or absence of genomic alterations in 11 genes that are associated with FDA-approved therapies (13). FDA-approved therapies are generally recommended by professional guidelines as first-line treatment of patients with therapy-associated alterations (13, 14). Patients with advanced NSCLC that harbor therapy-associated alterations have improved overall survival when treated with matched targeted therapies compared to chemoimmunotherapy (15, 16). For patients without FDA-approved targeted therapy options, clinical trial-associated alterations can inform alternative therapy options and clinical trial participation is encouraged by professional guidelines (13).

The increasing complexity of treatment decision-making for patients with advanced NSCLC necessitates broad molecular profiling before first-line therapy (13, 17). Current practice guidelines recommend establishing histologic subtypes with adequate tissue for biomarker testing. For patients with non-squamous NSCLC, molecular testing is recommended for genomic alterations in 11 genes (*EGFR, ALK, KRAS, ROS1, BRAF, NTRK1/2/3, MET*, *RET*, and *ERBB2*) in addition to PD-L1 immunohistochemistry (IHC) using a broad next-generation sequencing (NGS) panel to capture oncogenic driver alterations for matched FDA-approved therapies or clinical trials (13). For patients with squamous cell carcinoma, guidelines state molecular testing should be considered (13). Comprehensive genomic profiling (CGP) is a broad molecular profiling approach that utilizes NGS to detect known and novel alterations in hundreds of genes and immune signatures to inform treatment decisions across all solid cancer types. Some panels include RNA-seq which can increase the rates of gene fusion detection. As such in real-world clinical practice, CGP increases successful biomarker testing and helps avoid potentially missed treatment options in patients with newly diagnosed, advanced NSCLC (18).

Although biomarker-driven targeted therapy and immunotherapy have revolutionized the treatment landscape of metastatic NSCLC, clinicians must be cognizant of the precise sequencing of treatment and the potential development of resistance and/or co-occurring alterations that can hinder treatment responses. The effect of oncogenic drivers on immunotherapy efficacy is an area of active investigation (10, 17, 19). Although some patients with NSCLC achieve durable responses with immune checkpoint inhibitors (ICIs), not all patients benefit, and many tumors are resistant to treatment (19–21). Studies have demonstrated that upfront ICI monotherapy has low efficacy in patients with driver-positive NSCLC, although some exceptions exist (16, 17, 19). Another area of active clinical investigation is the impact of co-mutations on targeted therapy response (9, 22, 23). For example, recent investigations into factors that confer primary resistance to *KRAS* G12C inhibitors revealed a diversity of genomic resistance mechanisms, including co-mutations in *STK11, KEAP1*, and *TP53* (22, 24). Ongoing clinical trials with *KRAS* G12C inhibitors are investigating combination approaches to combat resistance (24, 25). Thus, understanding genomic heterogeneity and the interplay of co-occurring alterations in NSCLC tumors is becoming increasingly important in understanding responses to both targeted and immunotherapy.

Herein, we assessed the spectrum of current FDA therapy-associated and clinical trial-associated biomarkers, along with known and novel patterns of co-mutations and co-occurrence with biomarkers predictive of immunotherapy response, using the Illumina TruSight^®^ Oncology 500 (TSO 500) CGP assay (26). The TSO 500 is an analytically validated, broad-coverage CGP assay that uses DNA sequencing to detect small variants in the entire exonic coding region of 523 genes (single and multi-nucleotide substitutions, insertions, and deletions), copy number alterations in 59 genes (gains and losses), as well as analysis of microsatellite instability (MSI) and TMB genomic signatures. RNA sequencing is concurrently performed to detect fusions and splice variants in 55 genes. In this study, we perform the largest clinical study to date utilizing the TSO 500, providing an opportunity to further characterize the landscape of NSCLC and show its clinical utility in detecting known and novel facets of NSCLC.

## 2 Methods

### 2.1 Patient cohort

Approval for this study, including waiver of informed consent, was obtained from the Western Institutional Review Board Copernicus Group (WCG protocol # 1340120).

We retrospectively analyzed clinical CGP testing data from NSCLC FFPE tumor biopsy specimens submitted for CGP testing at a reference laboratory (OmniSeq/Labcorp, Buffalo, NY) during standard clinical care from June 2021 - June 2024. Specimens were collected from 647 provider facilities across the United States and Alaska. Any cases that were ultra-hypermutated (TMB > 200 mutations/Mb) and did not have adenocarcinoma or squamous cell carcinoma histology were excluded from the study. The total number of cases included in the study was 7,606 (5,523 with adenocarcinoma and 2,083 with squamous cell carcinoma).

### 2.2 Comprehensive genomic profiling

DNA and RNA were co-extracted from FFPE tissue specimens and submitted for library preparation and sequencing using the hybrid-capture-based TSO 500 assay (Illumina, San Diego, CA, USA) as part of OmniSeq^®^ INSIGHT (OmniSeq/Labcorp, Buffalo, NY, USA). OmniSeq^®^ INSIGHT is a comprehensive genomic and immune profiling assay performed in a laboratory accredited by the College of American Pathologists (CAP) and certified by the Clinical Laboratory Improvement Amendments (CLIA) (26). OmniSeq^®^ INSIGHT is an NGS-based *in vitro* diagnostic device for detecting genomic variants, signatures, and immune gene expression in FFPE tumor tissue. Within the OmniSeq^®^ INSIGHT framework, DNA sequencing with hybrid capture (via TSO 500) detects small nucleotide variants (SNVs) in exonic regions of 523 genes (single and multi-nucleotide substitutions, insertions, and deletions), copy number variants (CNVs) in 59 genes (gains and losses), as well as analysis of microsatellite instability (MSI) and TMB genomic signatures. RNA sequencing with hybrid capture (via TSO 500) detects fusions and splice variants in 55 genes. Variant annotation is performed using the GenomeOncology Precision Oncology Platform (GenomeOncology, Cleveland, OH, USA). Only genomic alterations annotated as known pathogenic were analyzed in the current study.

### 2.3 Immunohistochemical studies

For all tumor types, PD-L1 expression on the surface of tumor cells was measured by Dako PD-L1 IHC 22C3 pharmDx (Agilent, Santa Clara, CA). A board-certified anatomical pathologist scored expression according to published guidelines (27) as tumor proportion score (TPS), the percentage of tumor cells with positive linear membranous staining.

### 2.4 Statistical analysis

Statistical analysis and plot generation were performed in R v 4.4.1 (https://www.r-project.org/). All plotting was performed using the ggplot2 v 3.5.1 package (https://ggplot2.tidyverse.org/) and various packages to extend the ggplot2 functionality (ggpubr, ggtext, GGally, ggraph).

To assess differences between adenocarcinoma and squamous cell carcinoma NSCLC in patient and tumor characteristics and genomic alteration prevalence, Fisher’s exact test (via `fisher.test` function) or linear regression (via `lm` function) was performed for categorical or quantitative variables, respectively.

To assess differences in the distributions of immunotherapy markers in tumors with guideline-indicated alterations, non-guideline-indicated alterations, and tumors without alterations in any currently known driver genes, we tested for differences in immunotherapy markers between the different alteration groups in known NSCLC driver genes (*ALK*, *EGFR*, *MET*, *BRAF*, *ROS1*, *KRAS*, *ERBB2*). TMB was treated as a quantitative variable (mutations/Mb), and PD-L1 22C3 IHC TPS was treated as a categorical variable with levels of “Negative (<1%)”, “Low (1 - 49%)”, and “High (≥50%)”. Tests for differences in TMB and PD-L1 were performed using linear regression (via the `lm` function) and penalized likelihood ratio tests (via `logistf` and `logistftest` functions from the logistf v 1.26.0 package), respectively. Tests were adjusted for NSCLC histology by adding this variable as a covariate in the analysis. P-values were corrected for multiple testing within each driver gene using the `p.adjust` function with `method=“ bonferroni”`. Distributions of TMB and PD-L1 levels were plotted for alteration groups within each gene using ggplot2.

To detect co-occurrence or mutual exclusivity of genomic alterations, pairwise Fisher’s exact tests were performed between each pair of gene-level summarized genomic alterations using the `fisher.test` function. P-values were multiple testing corrected using the Benjamini-Hochberg false discovery rate (FDR) method as implemented in the `p.adjust` function when `method=“BH”`. Multiple testing corrected FDR q-values < 0.05 were considered significant. The proportion of co-occurrence between genomic alterations was also calculated to discern which pairs had complete exclusivity (i.e., the proportion of co-occurrence = 0). Only genomic alterations detected in at least 1% of patient tumors were included in the analysis.

To model higher-order co-occurrences or mutual exclusivities and detect gene modules of tightly co-occurring genomic alterations, network analysis was applied to co-occurrence results to generate a network of pairwise interactions between gene-level summarized genomic alterations. Co-occurrence analysis results were filtered for significant associations and then imported into the igraph v 1.3.5 package (https://igraph.org/) to create an igraph network. The degree and weighted degree for each node of the network (i.e., the genomic alterations and immunotherapy markers) were calculated using the `degree` and `strength` functions, respectively, from the graph specifying `weights` of the weighted degree to be the absolute odds ratio (OR) from Fisher’s exact test. The absolute OR was taken to be the OR if the OR was > 1 or the inverse of the OR if the OR < 1. Gene modules were defined within the network by how tightly their genomic alterations associated with one another using the community detection algorithm implemented in the `cluster_infomap` function in igraph, specifying the `e.weights` to be the ORs from Fisher’s exact test and the `v.weights` to be the weighted degree of each node. The R implementation of the Force Atlas 2 algorithm (from the ForceAtlas2 v 0.1 package, https://github.com/analyxcompany/ForceAtlas2) was used to position nodes in the network. The network was plotted using the graph v 2.1.0 package (https://bioconductor.org/packages/release/bioc/html/graph.html) specifying the Force Atlas 2 coordinates as the positions for the nodes.

To assess if a dose-dependent relationship existed between histology, immunotherapy markers (TMB, PD-L1), and an increasing number of genomic alterations within a gene module, differences in each variable were tested between NSCLC tumors with none, one, or ≥2 genomic alterations within genes co-occurring in each network-derived gene module.

## 3 Results

### 3.1 Patient and patient tumor characteristics

We retrospectively analyzed real-world CGP data from 7,606 patients with NSCLC who received CGP testing via TSO 500 and PD-L1 IHC at a reference laboratory during standard clinical care. Testing was performed on FFPE tumor biopsy specimens between 2021 and 2024. Specimens were collected from 647 provider facilities across the United States and Alaska. Patient and patient tumor characteristics in the full cohort and when stratified by histology subtype are provided in **Table 1**. Most of the cohort was lung adenocarcinomas (73%), with squamous cell carcinomas comprising the remaining third (27%). The mean age of patients was 70.7 ± 9.7 years, with 49% males and 51% females. A significantly higher frequency of females had tumors with adenocarcinoma, while males had significantly more tumors with squamous cell histology (P=4E-35; **Table 1**). Tissue specimens for this study came from the primary tumor site in 70.4% of cases and the advanced or metastatic site in 18.9% and 10.8% of cases, respectively, with adenocarcinoma cases being significantly enriched in the latter (P<3E-21; **Table 1**). For patients with known staging information (N=2,631), 16% were stage III and 66.2% were stage IV. The mean number of detected pathogenic alterations for all NSCLC cases was 3.9 ± 2.4 with adenocarcinoma cases having significantly less on average than squamous cell carcinoma (3.8 ± 2.3 vs 4.2 ± 2.5, P=7E-8; **Table 1**). The mean TMB level for all NSCLC cases was 11 ± 11.1 mutations/Mb, with 12.5% of cases having a TMB of ≥20 mutations/Mb, 30.2% having a TMB of 10-19 mutations/Mb, and 57.2% having a TMB of <10 mutations/Mb. The mean PD-L1 TPS score among tumors was 22.2 ± 31.1, with 25.1% of cases having a TPS of ≥50%, 37.7% having a TPS of 1 - 49%, and 37.2% having a TPS of <1%. Most cases were microsatellite stable, with only 23 MSI high cases (0.3%). Tumors with adenocarcinoma histology tended to have more cases with TMB of <10 mutations/Mb (P=1E-22) and negative PD-L1 (P=5E-8) compared to squamous cell carcinoma cases, however, showed a slight, but significant, enrichment of tumors with high/very high levels of these markers (P<0.02; **Table 1**).

**Table 1:** Patient and patient tumor characteristics.

Variable	All patients		Adenocarcinoma		Squamous cell carcinoma		P
	N	Summary stats	N	Summary stats	N	Summary stats	
**Total number of patients**	7606	–	5523	–	2083	–	–
**Gender (N, %)**	7603		5521		2082		
Female		3881 (51%)		3058 (55.4%)		823 (39.5%)	
Male		3722 (49%)		2463 (44.6%)		1259 (60.5%)	4E-35
**Age (Mean ± SD)**	7606	70.7 ± 9.7	5523	70.4 ± 10	2083	71.5 ± 8.7	7E-6
**Age group (N, %)**	7606		5523		2083		
≤40		44 (0.6%)		39 (0.7%)		5 (0.2%)	0.017
41-50		153 (2%)		136 (2.5%)		17 (0.8%)	1E-6
51-60		832 (10.9%)		653 (11.8%)		179 (8.6%)	4E-5
61-70		2589 (34%)		1854 (33.6%)		735 (35.3%)	0.17
71-80		2758 (36.3%)		1940 (35.1%)		818 (39.3%)	8E-4
81-90		1145 (15.1%)		837 (15.2%)		308 (14.8%)	0.72
>90		85 (1.1%)		64 (1.2%)		21 (1%)	0.63
**Tissue specimen location (N, %)**	7606		5523		2083		
Primary		5352 (70.4%)		3618 (65.5%)		1734 (83.2%)	4E-55
Advanced		1436 (18.9%)		1203 (21.8%)		233 (11.2%)	7E-28
Metastatic		818 (10.8%)		702 (12.7%)		116 (5.6%)	3E-21
**Unknown clinical stage (N, %)**		4975 (65.4%)		3513 (63.6%)		1462 (70.2%)	–
**Known clinical stage (N, %)**		2631 (34.6%)		2010 (36.4%)		621 (29.8%)	–
**Known clinical stage (N, %)**	2631		2010		621		
Stage I		301 (11.4%)		246 (12.2%)		55 (8.9%)	0.021
Stage II		166 (6.3%)		109 (5.4%)		57 (9.2%)	1E-3
Stage III		422 (16%)		262 (13%)		160 (25.8%)	5E-13
Stage IV		1742 (66.2%)		1393 (69.3%)		349 (56.2%)	3E-9
**Number of detected known pathogenic alterations (Mean ± SD)**	5781	3.9 ± 2.4	4190	3.8 ± 2.3	1591	4.2 ± 2.5	7E-8
**Genomic variants with known or potential clinical significance (N, %)**	7606		5523		2083		
Guideline-indicated		3328 (43.8%)		3176 (57.5%)		152 (7.3%)	0
Clinical trial or therapy in other tumor type		5887 (77.4%)		4385 (79.4%)		1502 (72.1%)	3E-11
Neither above, but known pathogenic		1641 (21.6%)		1064 (19.3%)		577 (27.7%)	5E-15
**TMB (Mut/Mb) (Mean ± SD)**	6705	11 ± 11.1	4865	10.7 ± 11.3	1840	11.8 ± 10.6	2E-4
**TMB level (N, %)**	6705		4865		1840		
Very high (≥20)		841 (12.5%)		645 (13.3%)		196 (10.7%)	4E-3
High (10-19)		2027 (30.2%)		1258 (25.9%)		769 (41.8%)	1E-35
Not high (<10)		3837 (57.2%)		2962 (60.9%)		875 (47.6%)	1E-22
**PD-L1 22C3 TPS (Mean ± SD)**	7558	22.2 ± 31.1	5487	22.7 ± 31.5	2071	21.1 ± 29.8	0.049
**PD-L1 level (N, %)**	7558		5487		2071		
High (≥50%)		1897 (25.1%)		1416 (25.8%)		481 (23.2%)	0.022
Low (1 - 49%)		2848 (37.7%)		1927 (35.1%)		921 (44.5%)	1E-13
Negative (<1%)		2813 (37.2%)		2144 (39.1%)		669 (32.3%)	5E-8
**MSI level (N, %)**	6708		4863		1845		
MSI High		23 (0.3%)		9 (0.2%)		14 (0.8%)	
Stable		6685 (99.7%)		4854 (99.8%)		1831 (99.2%)	1E-3

N, number of cases with data for variable; SD, the standard deviation of the mean; TMB, tumor mutational burden, measured as mutations per megabase (Mut/Mb); TPS, tumor proportion score (i.e., the proportion of tumors positive for PD-L1 immunohistochemistry with 22C3 antibody as observed by a pathologist); MSI, microsatellite instability score; P, uncorrected P-value from testing differences between adenocarcinoma and squamous cell carcinoma using Fisher’s exact test or linear regression for categorical or quantitative variables, respectively.

### 3.2 Prevalence of pathogenic genomic alterations detected by TSO 500

**Figure 1:**
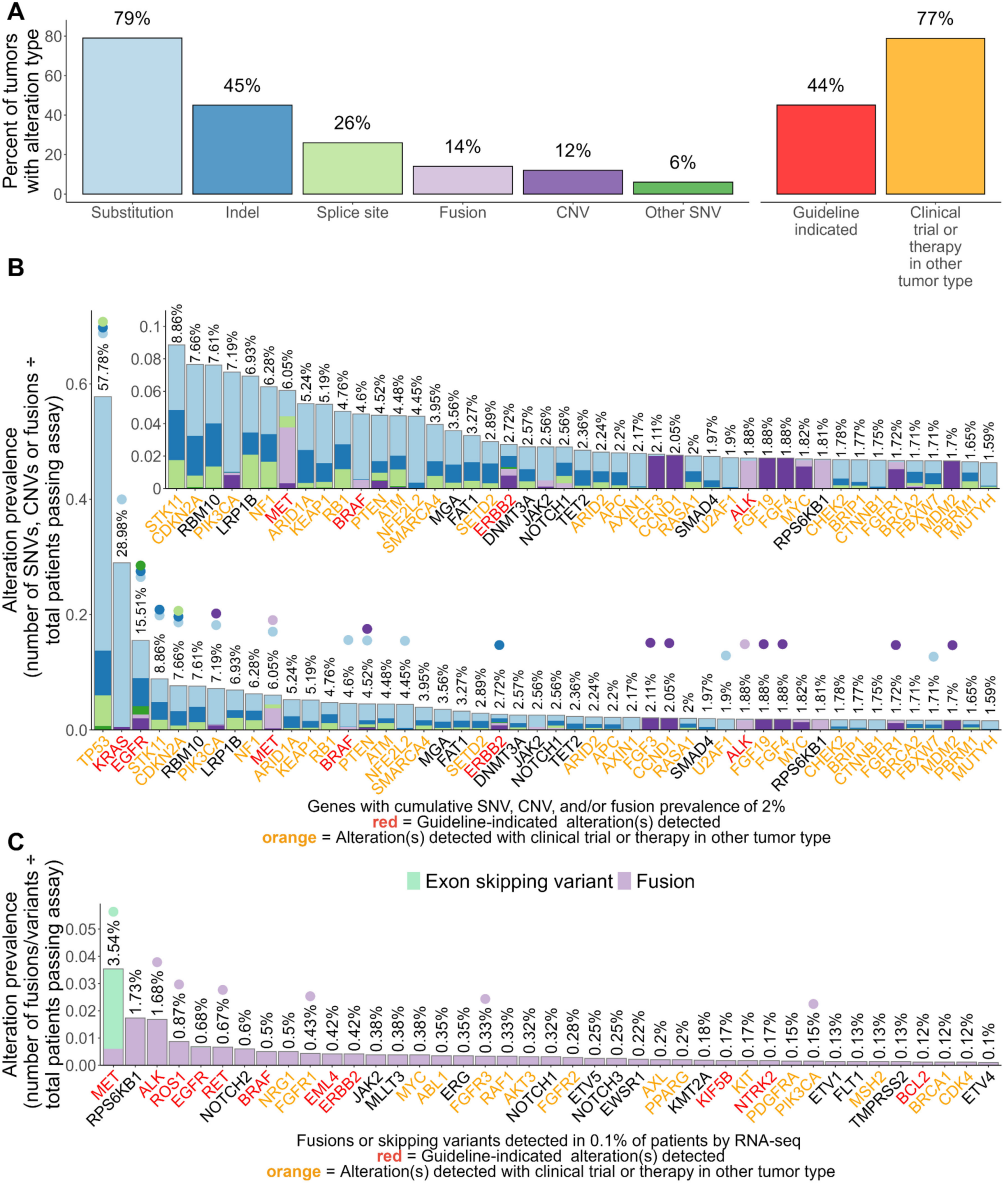
Landscape of genomic alterations detected by TSO 500 in 7,606 NSCLC tumors. **(A)** Overall prevalence of alteration types detected including clinically informative alterations. **(B)** Gene-level summarized prevalence of detected genomic alterations. **(C)** Gene-level summarized prevalence of fusions and exon skipping mutations detected via RNA components of the TSO 500 assay. For **(B, C)**, the Y-axis shows the prevalence of alterations, which are visualized via a stacked bar plot and summarized at the gene level. Prevalence was calculated by dividing the number of patient tumors with detected SNVs, CNVs, or fusions/skipping variants by the total number of patient specimens that passed the corresponding assay component. Percentages on top of each stacked bar represent the cumulative prevalence of all alterations for a gene. Points were placed above percentages to note what genomic alterations within each gene had significantly different prevalences between adenocarcinoma and squamous cell carcinoma. The X-axis shows genes whose combined alterations were detected in ≥2% **(B)** or ≥0.1% **(C)** of patient tumors. Genes are sorted based on their combined alteration prevalence in patient tumors. Gene names are colored red or orange if a guideline-indicated alteration or alteration matched to a clinical trial or therapy in another tumor type was detected within the gene for one or more patient tumors. A zoomed-in panel within the main stacked bar plot of **(B)** shows the prevalence of alterations for genes whose cumulative alteration prevalence was <10%.

Of the 7,606 samples tested, 44% were positive for therapy-associated genomic alterations, and 77% were positive for clinical trial-associated genomic alterations or genomic alterations with an accepted therapy in another tumor type (**Figure 1A**; **Table 1**). Adenocarcinomas harbored significantly more therapy- (57.5%) and clinical trial- (79.4%) associated variants than squamous cell carcinomas (7.3% and 72.1%, respectively) (P<3E-11; **Table 1**). The most prevalent genomic alterations detected in NSCLC tumors were substitutions (79%) followed by small insertions/deletions (Indels; 45%), splice site variants (26%), gene fusions (14%), larger CNVs (12%), and other complex types of SNVs (6%) (**Figure 1A**).

Pathogenic alterations in 296 genes were detected in ≥0.1% of tested tumors with alterations in *TP53*, *KRAS*, or *EGFR* being detected in >10% of tested tumors (**Figure 1B**; **Supplementary Table S1**). Adenocarcinoma cases had a higher prevalence of SNVs, CNVs, and/or fusions/rearrangements in 28 genes including therapy-associated genes such as *KRAS* (37.5% vs 4.6%), *EGFR* (17.1% vs 1.3%), *BRAF* (5.2% vs 1%), *ERBB2* (1.8% vs 0.8%), *RET* (0.9% vs 0.3%), and *ROS1* (1.2% vs 0.1%) (**Figure 1B**; **Supplementary Table S1**). Squamous cell carcinomas had a significantly higher prevalence of genomic alterations in 43 genes including *TP53* (81.9% vs 48.7%), *CDKN2A* (16.3% vs 4.4%), *PIK3CA* (11.6% vs 4.2%), *RB1* (6.6% vs 4.1%), and *NFE2L2* (13% vs 1.2%) (**Figure 1B**; **Supplementary Table S1**). FDA-emerging biomarkers were present in both histologies, with genes like *STK11*, *KEAP1*, and others more frequently altered in lung adenocarcinoma (**Figure 1B**; **Supplementary Table S1**). The most prevalent alterations identified by RNA sequencing included fusions or exon skipping mutations involving therapy-associated genes *MET* (2.9%), *ALK* (1.7%), *ROS1* (0.9%), and *RET* (0.7%), all of which were detected at a significantly higher frequency in adenocarcinoma cases (**Figure 1C**; **Supplementary Table S1**). Of note, squamous cell carcinoma cases had enrichment of fusions involving *FGFR1* (0.9% vs 0.3%), *FGFR3* (0.8% vs 0.2%), and *PIK3CA* (0.5% vs <0.01%) (**Figure 1C**; **Supplementary Table S1**).

In total, 399 specific mutations were detected at a prevalence of ≥0.1% among tested NSCLC tumors (**Supplementary Table S2**). The most prevalent single variants detected were therapy-associated substitutions in *KRAS* including G12C (11.3%), G12V (5.8%), G12D (4.2%), and G12A (2%). First-line therapy-associated variants in *EGFR* and *BRAF* were also among the topmost prevalent single variants including the *EGFR* L858R substitution (4.6%) and E746-A750 deletion (3.3%) and the *BRAF* V600E substitution (1.5%). These and 28 other mutations were detected at a significantly higher prevalence in adenocarcinoma cases including therapy-associated *MET* exon 14 skipping mutations (3.8% vs 0.8%), *EML4-ALK* fusions (2% vs 0%), and *KIF5B*-*RET* fusions (0.5% vs 0.1%) (**Supplementary Table S2**). Squamous cell carcinoma cases were enriched for 54 specific mutations compared to adenocarcinoma cases including several substitutions in *PIK3CA, TP53*, and *NFE2L2* that had current clinical trials in NSCLC or had an approved therapy in another tumor type (**Supplementary Table S2**). The most significantly enriched mutations, however, were amplifications of *PIK3CA* (2.9% vs <0.01%) and fibroblast growth factor (FGF)-associated genes including *FGF4* (5% vs 0.7%)*, FGF3* (5.2% vs 0.8%)*, CCND1* (5.2% vs 0.8%)*, FGF19* (4.7% vs 0.8%), and *FGFR1* (3.8% vs 0.2%), some of which (*PIK3CA, FGFR1)* had current clinical trials in NSCLC (**Supplementary Table S2**).

### 3.3 Associations between biomarkers of ICI response and oncogenic drivers in NSCLC

**Figure 2:**
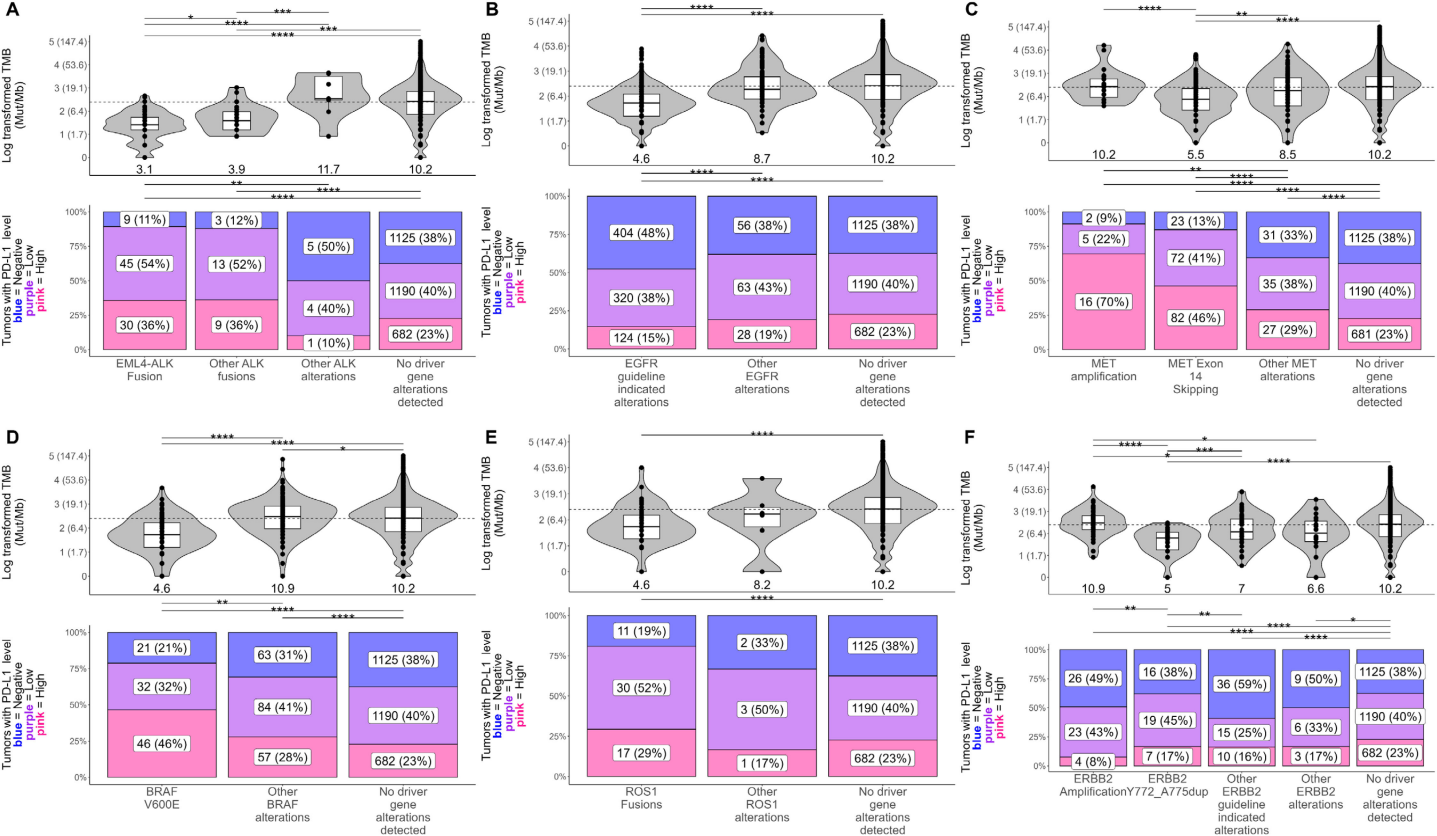
Associations between the presence of known non-KRAS driver mutations, TMB, and PD-L1 in NSCLC tumors. Differences in tumor mutational burden (TMB; in mutations/Mb) and PD-L1 tumor proportion score (TPS) were tested between NSCLC tumors with known driver/non-driver mutations within **(A)** *ALK*, **(B)** *EGFR*, **(C)** *MET*, **(D)** *BRAF*, **(E)** *ROS1*, and **(F)** *ERBB2*. For plots of TMB (top), each dot in the box-violin plots represents an individual tumor from a unique patient falling into a particular variant group (X-axis) and is plotted along the Y-axis based on its log-transformed TMB value (non-transformed TMB value of each transformed value shown in parentheses on the Y-axis for clarity). The bottom, middle, and top horizontal boundaries of each box in the box-violin plots represent the first, second (median), and third quartiles of the data. The lines extending from the two ends of each box represent 1.5x outside the interquartile. Points beyond the lines are considered outliers. The width of the grey-shaded regions around the boxes represents the density of the data points, where wider areas correspond to higher data point density. Values listed under the box-violin plots are the median for the group. For plots of PD-L1 TPS level (bottom), each section of the bar plots represents the percent of tumors falling in a particular variant group (X-axis) that were deemed to be negative (<1%), low (1 - 49%), or high (≥50%) for PD-L1 TPS. Differences in TMB and PD-L1 between groups were tested using linear regression and penalized likelihood ratio tests, respectively, adjusting for NSCLC histology. Results of testing can be found in **Supplementary Table S4**. *, P<0.05; **, P<0.01; ***, P<0.001; ****, P<0.0001.

We next examined the association between TMB and PD-L1 expression and oncogenic driver mutations in known driver genes of NSCLC including *ALK*, *EGFR*¸ *MET, BRAF, ROS1, ERBB2*, and *KRAS*. To detect associations, we tested for differences in TMB and PD-L1 between tumors with known driver mutations compared to tumors with non-driver mutations in the same gene and tumors without any alterations in any known driver genes of NSCLC (driver gene negative) (**Figure 2**; **Supplementary Figure S1**; **Supplementary Tables S3**, **S4**).

Overall, significantly lower TMB levels were observed across all tumors with therapy-associated driver mutations and significantly higher PD-L1 levels were observed in tumors with *ALK*, *MET*, *BRAF*, or *ROS1* driver mutations (**Figure 2**; **Supplementary Table S4**). Tumors with *EML4-ALK* and other *ALK* fusions had a significantly lower median TMB (3.1 mut/Mb; 3.9 mut/Mb) compared to those with non-fusion *ALK* alterations (11.7 mut/Mb) and driver gene negative tumors (10.2 mut/Mb) yet had a significantly higher frequency of high PD-L1 TPS scores (scores ≥50%) than the other tumor groups (36% vs ≤23%) (**Figure 2A**). Tumors harboring therapy-associated *EGFR* alterations had significantly lower TMB (median 4.6 mut/Mb) and frequency of high PD-L1 scores (15%) compared to tumors with other *EGFR* alterations and driver-gene negative tumors (≥9 mut/Mb and ≥19%) (**Figure 2B**). Tumors with *MET* exon 14 skipping mutations had a significantly lower TMB (5.5 mut/Mb) compared to tumors with *MET* amplifications, other *MET* alterations, and driver gene negative tumors (≥8.5 mut/Mb) (**Figure 2C**). Tumors with *MET* exon 14 skipping and *MET* amplifications had significantly higher frequencies of high PD-L1 scores (46% and 70%) compared to tumors with other *MET* alterations and driver gene-negative tumors (≤29%) (**Figure 2C**). Of note, *MET-*amplified tumors had the highest frequency of high PD-L1 scores out of all tested tumor groups, with most of these tumors being detected as PD-L1 high (**Figure 2C**). Tumors with *BRAF* V600E mutations had significantly lower TMB scores (4.6 mut/Mb) compared to other *BRAF* alterations and driver gene negative tumors (≥10.2 mut/Mb) yet had a significantly higher frequency of high PD-L1 scores (46%) than the other tumor groups (≤28%) (**Figure 2D**). Similarly, tumors with *ROS1* fusions had significantly lower TMB (4.6 mut/Mb) than driver gene-negative tumors, but significantly higher frequency of high PD-L1 scores (29%) (**Figure 2E**). Tumors with *ERBB2* exon 20 insertions (Y772-A775dup) had significantly lower TMB (5 mut/Mb) than driver gene-negative tumors and tumors with *ERBB2* amplifications or other therapy-associated *ERBB2* alterations (≥7 mut/Mb) (**Figure 2F**). Tumors with *ERBB2* amplifications or other therapy-associated *ERBB2* alterations had significantly higher frequencies of PD-L1 negative scores (≥49%) compared to driver gene negative tumors (38%) (**Figure 2F**).

Comparing different *KRAS* alterations, tumors with *KRAS* G12D (6.2 mut/Mb), G12A (8.6 mut/Mb), G12V (8.6 mut/Mb), and Q61H (7 mut/Mb) alterations had the lowest TMB among tumors with *KRAS* alterations (<10 mut/Mb) and significantly lower TMB than all other tested *KRAS* tumor groups (≥10 mut/Mb) (**Supplementary Figure S1A**; **Supplementary Table S4**). Tumors with *KRAS* G13C and G13D had the highest frequency of PD-L1 scores ≥50% (≥41%) among tumors with *KRAS* alterations and had a significantly higher frequency than tumors positive for Q61H and driver gene negative tumors (≤23%) (**Supplementary Figure S1B**; **Supplementary Table S4**). Tumors with *KRAS* Q61H had the lowest frequency of high PD-L1 scores (18%) and was the only group of *KRAS*-altered tumors that did not exhibit significantly different PD-L1 levels from driver gene-negative tumors (**Supplementary Figure S1B**; **Supplementary Table S4**).

### 3.4 Co-occurring and mutually exclusive genomic alterations

**Figure 3:**
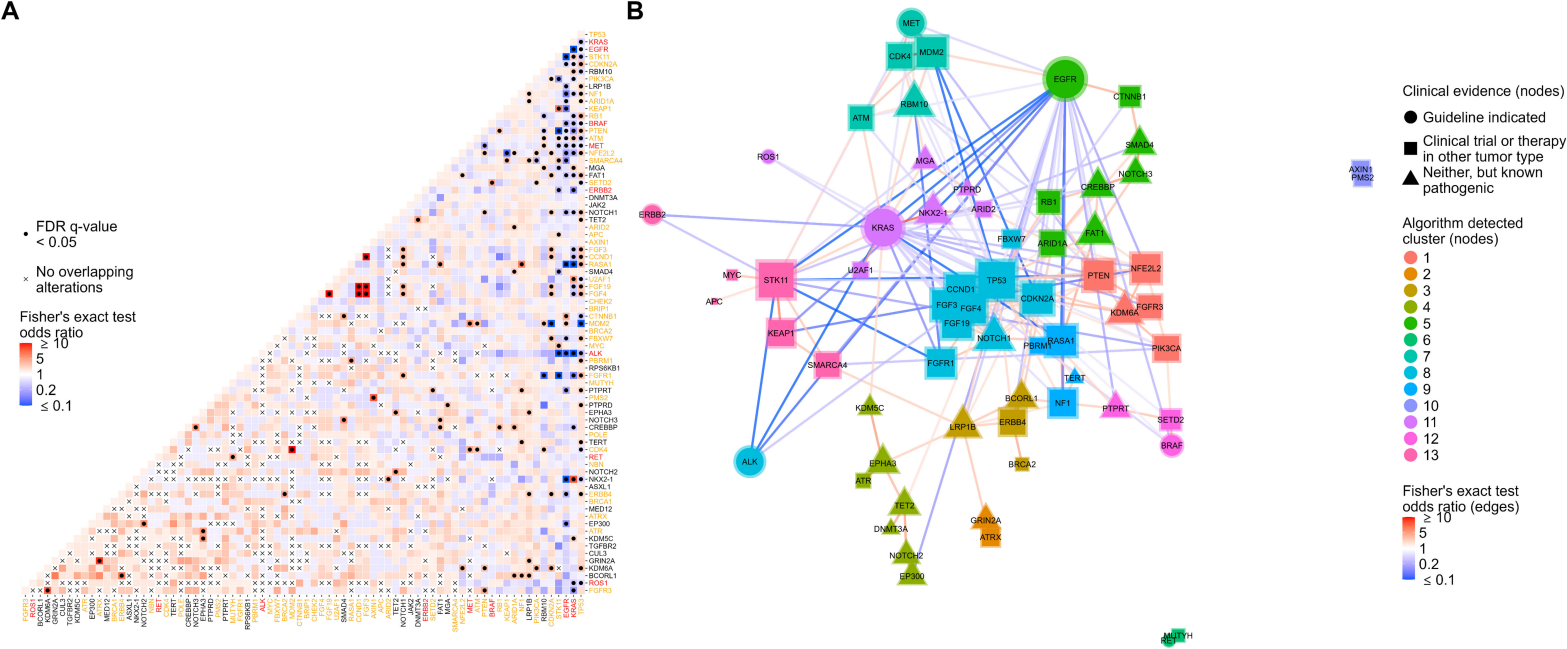
Co-occurrence analysis followed by network analysis with gene module detection to assess co-occurrences and mutual exclusivities between genomic alterations in NSCLC. **(A)** Co-occurrence or mutual exclusivity of genomic alterations were assessed at the gene level using pairwise Fisher’s exact tests and visualized via heatmap where red cells indicate co-occurrence (Fisher’s exact test odds ratio > 1), and blue cells represent mutual exclusivity (odds ratio < 1). Cells marked with a dot indicate co-occurrences/mutual exclusivities that reached significance (multiple testing corrected false discovery rate q-value < 0.05). Cells marked with an “x” indicate complete mutual exclusivity (i.e., no alterations were found in the same tumor for those genes). Only genes with alterations detected in at least 1% of patient tumors were included in the analysis. Gene names are colored red or orange if a guideline-indicated alteration or alteration matched to a clinical trial or therapy in another tumor type was detected within the gene for one or more patient tumors. **(B)** Odds ratios of significant co-occurrences/mutual exclusivities were extracted and used to construct a gene network where red and blue connecting lines denote a co-occurring or mutually exclusive association, respectively. The shape of nodes in the network relates to the highest clinical evidence of genomic alterations detected in each gene and each node is sized based on the number and strength of its connections. A community detection algorithm was used to detect gene modules within the network, and each node was colored based on its module membership. Nodes in the networks were positioned using the force-directed algorithm ForceAtlas2.

Evaluation of co-occurring genomic alterations in NSCLC revealed co-mutational patterns including alterations with and without associated targeted therapies and clinical trials (**Figure 3A**; **Supplementary Table S5**). Genes with therapy-associated alterations were mainly mutually exclusive, with *KRAS* and *EGFR* having the most significant mutual exclusivities with other genes (23 each) (**Figure 3A**; **Supplementary Table S5**). In contrast, genes with clinical trial-associated alterations or alterations with therapy in another tumor type had more significant co-occurrences, with *TP53* having the most of all tested genes (23 co-occurring genes) (**Figure 3A**; **Supplementary Table S5**). The highest proportion of co-occurrence (i.e., percent of tumors that harbored genomic alterations from a pair of genes) was observed between genes involved in the FGF pathway (*FGF3/4/19, CCND1*; 80% - 87%) followed by *MDM2* and *CDK4* (27%) and *STK11* and *KEAP1* (15%) (**Supplementary Table S5**).

To further investigate significantly co-occurring and mutually exclusive genomic alterations (FDR q-value < 0.05), we performed network analysis followed by gene module detection to model higher-order interactions between genomic alterations and detect modules of tightly associated genes (**Figure 3B**). Network analysis highlighted the mutual exclusivity of essential driver genes in NSCLC including *EGFR*, *KRAS*, *BRAF*, *ALK*, and *MET* (**Figure 3B**). A tight cluster was formed in the network between *CCND1, FGF19, FGF4*, and *FGF3*, which mirrors our previous observation of them having the highest proportions of co-occurrence than any other groups of genes (**Figure 3B**). Genes in the network were grouped into 13 modules of tightly associating genes (including co-occurring and mutually exclusive genes) (**Figure 3B**). Most gene modules included a mix of genes with clinical trial-associated alterations or alterations with therapy in another tumor type and genes with pathogenic alterations of unknown clinical significance. Interestingly, if a gene module included a gene with therapy-associated alterations, it typically was the only therapy-associated gene in the module, reinforcing their mutual exclusivity to other genes with therapy-associated alterations (**Figure 3B**).

### 3.5 Dose-dependent relationships between the number of genomic alterations in a gene module, histology, and biomarkers of ICI response

**Figure 4:**
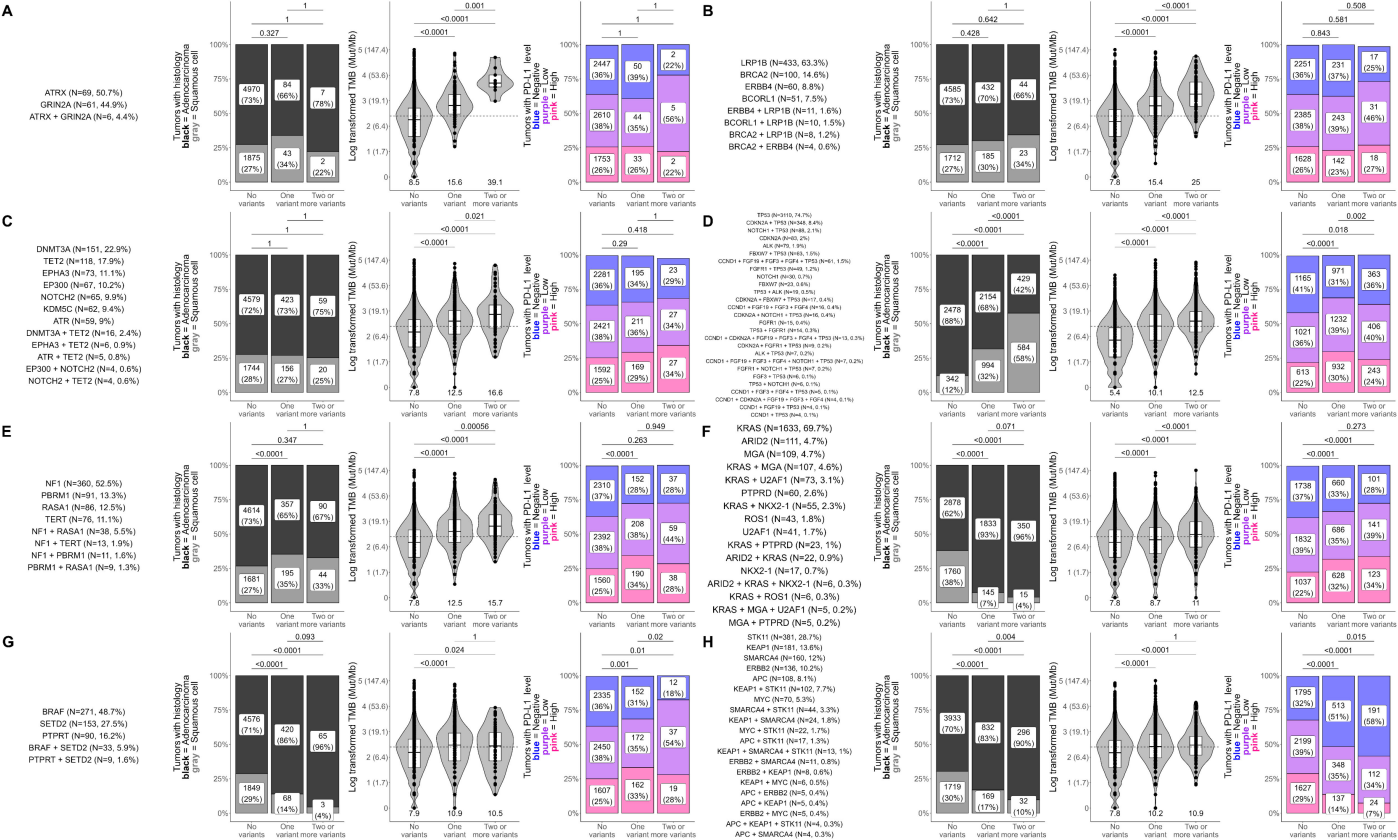
Dose-dependent influences of gene module alteration number on histology, TMB, and PD-L1 in NSCLC. Differences in histology, tumor mutational burden (TMB; in mutations/Mb), and PD-L1 tumor proportion score (TPS) were tested between NSCLC tumors with none, one, or ≥2 genomic alterations within genes co-occurring in each network-derived gene module. Analyses were performed for all gene modules and results for **(A)** *ATRX/GRIN2A*, **(B)** *LRP1B/BRCA*, **(C)** *DNMT3A/TET2*, **(D)** *TP53*, **(E)** *NF1*, **(F)** *KRAS*, **(G)** *BRAF*, and **(H)** *STK11/KEAP1 -* associated gene modules are shown here. Results for all other gene modules are presented in **Supplementary Figure S2**. The first column for each gene module shows the unique combinations of genes that had co-occurring alterations in the gene module. For clarity, only gene combinations detected in at least 4 patient tumors are shown. The remaining plots show differences in the distribution of histology (adenocarcinoma vs squamous cell carcinoma), TMB, and PD-L1 between tumors that had none, one, or ≥2 genomic alterations in genes of the gene module. For plots of TMB (third column), each dot in the box-violin plots represents an individual tumor from a unique patient falling into a particular variant group (X-axis) and is plotted along the Y-axis based on its log-transformed TMB value (non-transformed TMB value of each transformed value shown in parentheses on the Y-axis for clarity). The bottom, middle, and top horizontal boundaries of each box in the box-violin plots represent the first, second (median), and third quartiles of the data. The lines extending from the two ends of each box represent 1.5x outside the interquartile. Points beyond the lines are considered outliers. The width of the grey-shaded regions around the boxes represents the density of the data points, where wider areas correspond to higher data point density. Values below each box-violin plot is the untransformed median TMB of the group. For plots of PD-L1 TPS level (last column), each section of the bar plots represents the percent of tumors falling in a particular variant group (X-axis) that were deemed to be negative (<1%), low (1 - 49%), or high (≥50%) for PD-L1 TPS. Differences in histology, TMB, and PD-L1 were tested using Fisher’s exact test, t-test, and chi-squared test, respectively.

To further characterize the relationships between co-occurring genomic alterations in detected gene modules and clinically important factors of NSCLC including histology and biomarkers of ICI response (TMB, PD-L1), we tested for dose-dependent associations between these factors and an increasing number of genomic alterations within a gene module (**Figure 4**; **Supplementary Figure S2**). For each detected gene module, we calculated the frequencies of each unique combination of genes with co-occurring alterations (including single genes) and labeled each gene module by the gene(s) with the highest frequencies (**Figure 4**; **Supplementary Figure S2**, first column). This resulted in gene modules being designated the following: *ATRX/GRIN2A*, *LRP1B/BRCA*, *DNMT3A/TET2, TP53*, *NF1*, *KRAS*, *BRAF, STK11*/*KEAP1*, *PIK3CA*, *EGFR, MUTYH/RET, RBM10*/*MET*, and *AXIN1* – associated gene modules. We then tested differences in histology, TMB, and PD-L1 between tumors with none, one, or ≥2 genomic alterations within genes co-occurring in each network-derived gene module (**Figure 4**; **Supplementary Figure S2**, columns 2-4).

Out of the 11 gene modules, 8 showed a clear dose-dependent association between an increasing number of genomic alterations and TMB or PD-L1 (**Figure 4**). Results for the remaining gene modules can be found in **Supplementary Figure S2**.

Significant associations between increasing numbers of genomic alterations and TMB were observed for *ATRX/GRIN2A, LRP1B/BRCA, DNMT3A/TET2, TP53, NF1*, and *KRAS*-associated gene modules (P ≤ 0.02), starting at a median TMB below 10 mut/Mb and climbing to medians above 10 mut/Mb (**Figures 4A–F**). The most dramatic increases were observed in the *ATRX/GRIN2A, LRP1B/BRCA*, and *DNMT3A/TET2*-associated gene modules, reaching upwards of 50 mut/Mb for tumors with ≥2 genomic alterations (**Figures 4A–C**). Of note, the *DNMT3A*/*TET2*-associated gene module consists of genes involved in clonal hematopoiesis, so this association may not be directly related to changes in the tumor itself.

For PD-L1, increasing number of genomic alterations in the *BRAF*-associated gene module was associated with higher frequencies of PD-L1 positive tumors (P ≤ 0.02; **Figure 4G**), while increasing number of genomic alterations in the *STK11/KEAP1*-associated gene module was associated with lower frequencies of PD-L1 positive tumors (P ≤ 0.02; **Figure 4H**). The *KRAS*-associated gene module also showed a significant association with PD-L1 levels; however, no difference was found between tumors with one or ≥2 genomic alterations within this gene module suggesting that the more prevalent *KRAS* alterations were driving this signal as additional genomic alterations within this module did not affect PD-L1 levels (**Figure 4F**).

In addition to the TMB and PD-L1 associations, we observed significant associations between increasing numbers of genomic alterations and higher frequencies of squamous cell carcinoma within the *TP53* and *PIK3CA*-associated gene modules (P<0.0001; **Figure 4D**; **Supplementary Figure S2A**). *KRAS, BRAF, STK11*/*KEAP1, MUTYH/RET*, and *RBM10/MET*-associated modules showed the opposite trend, having significantly higher frequencies of adenocarcinoma cases as genomic alterations increased (P ≤ 0.02; **Figures 4F–H**; **Supplementary Figures S2C, D**).

## 4 Discussion

Comprehensive biomarker testing using NGS is recommended at diagnosis for patients with advanced or metastatic NSCLC to identify driver alterations with targeted therapies available or under investigation in clinical trials (13). Here, we retrospectively analyzed the CGP results of over 7,000 NSCLC tumors from patients who received testing with the TSO 500 CGP assay during routine clinical care to further characterize the landscape of NSCLC using this newer technology and show its clinical utility in detecting both known and novel facets of NSCLC. Utilizing the broad coverage of the TSO 500, we were able to provide (1) a comprehensive view of the landscape and prevalence of treatment- or clinical trial-associated genomic alterations across NSCLC (**Figure 1**; **Supplementary Tables S1, S2**), (2) show associations of known driver mutations with biomarkers of ICI response (**Figure 2**; **Supplementary Tables S3, S4**), and (3) model higher-order interactions between co-occurring and mutual exclusive genomic alterations (**Figure 3**; **Supplementary Table S5**). These findings recapitulate previous findings (9) (14, 28), and add additional insights with regards to associations between driver gene mutations in tumors with very high PD-L1 expression (TPS ≥ 50%) or TMB (TMB ≥20) (**Figure 2**; **Supplementary Tables S3, S4**). Additionally, we identified modules of tightly associated genes and characterized dose-dependent relationships between the increasing number of genomic alterations within these modules, histology, and biomarkers of ICI response (**Figure 4**). While gene module detection and characterization in NSCLC has been attempted previously using RNA-seq (29–38), it has yet to be done using genomic results from a broad coverage CGP assay such as the TSO 500.

For the remainder of the discussion, we summarize our findings in the context of current clinical evidence to further show the clinical utility of performing CGP testing using targeted, broad coverage assays, such as the TSO 500, and highlight novel findings with lesser-known clinical evidence.

### 4.1 Mutual exclusivity of therapy-associated alterations

Mutual exclusivity of strong oncogenic driver gene variants (i.e. *KRAS*, *EGFR*, *ALK*) in NSCLC tumors is well described (39) and was observed in the current study. Identification of this driver oncogene in a patient’s tumor is critical to ensuring they receive the correct therapy. In a recent study, overall survival was significantly compromised in NSCLC patients who received genomic test results after starting first-line therapy compared to those who received results before starting on the appropriate matched therapy (40). Overall survival was also impacted in patients whose treatment was initiated before receiving results and then switched to the matched targeted therapy once the corresponding driver gene variant was identified. This further enforces the importance of obtaining CGP results before first-line therapy to identify the true driver mutation and matched targeted therapy.

### 4.2 Co-occurring gene alterations and potential resistance

We confirmed gene alterations that have significant co-occurrence with key therapy-associated genes and created a network of genes across the entire NSCLC cohort to model higher-order interactions in a novel way (**Figure 3B**). Several closely associated genes were identified in the current study that have been previously described across multiple individual analyses with varying levels of evidence (9). However, less is known about the clinical significance of other gene-to-gene associations we observed including co-occurring alterations in *MDM2*, *CDK4*, and *MET* (41) or the impact of co-occurring *CTNNB1* and *EGFR* alterations that may contribute to resistance to tyrosine kinase inhibitors (TKIs) (42, 43) (**Figure 3**; **Supplementary Table S5**).

### 4.3 Therapy-associated oncogenic driver alterations, PD-L1 expression, and TMB

#### 4.3.1 *EGFR* alterations and resistance to ICI

There are varying levels of evidence to describe associations between oncogenic driver gene mutations and biomarkers predictive of ICI benefit in patients with NSCLC, including PD-L1 expression and tumors with high TMB. Several reports have shown that *EGFR*-driven NSCLC tumors are associated with low median TMB levels and a higher percentage of tumors with low (TPS 1 - 49%) or negative PD-L1 expression, resulting in a cold immune microenvironment and lack of response to ICI therapy (16, 22). We also observed that *EGFR*-driven tumors are less frequently PD-L1 positive and have a lower median TMB as compared to tumors with no mutations in any driver gene (**Figure 2B**; **Supplementary Table S4**).

#### 4.3.2 Variable associations of ICI biomarkers in *KRAS* mutant tumors based on variant type and co-mutations

In our analysis, NSCLC tumors with *KRAS* mutations are associated with a higher frequency of PD-L1 expression (TPS 1 - 49%) compared to other driver mutations, except for *BRAF* V600E and *MET* exon 14 skipping mutations, and mutation-dependent associations with TMB (**Figures 2C, D**; **Supplementary Figure S1**), consistent with previous findings (22). The prevalence of NSCLC patients with tumors having PD-L1 expression TPS ≥50% or high TMB was previously shown to be significantly higher with *KRAS* G12C mutations compared to all other *KRAS* mutations (G12A/D/V, G13, Q61), with the highest prevalence of TMB high seen in tumors with G13 mutations and lowest in G12D tumors (44). Interestingly, our analysis found *KRAS* G13C/D tumors to have the highest frequency of PD-L1 TPS ≥50% (42% and 41%, respectively) with *KRAS* G12C tumors following next (38%), having no significant difference in PD-L1 scores (**Supplementary Figure S1B**). We also observed a lower median TMB for G12D tumors (6.2 mut/Mb) compared to all other *KRAS* mutations (i.e., *KRAS* G12C median TMB = 10.1 mut/Mb) (**Supplementary Figure S1A**). These findings are aligned with a previous study that also observed significantly decreased immune cell infiltration that was magnified in tumors with *KRAS* G12D and *TP53* mutations, implying this co-mutation signature could be a negative predictor of ICI benefit (45). *KRAS* G12D tumors are also more frequent in never or light smokers whereas *KRAS* G12C frequency is highest among current smokers, which can further explain the significant difference in median TMB between these subtypes that we, and others, have observed (44).

Identifying heterogeneous subtypes of *KRAS* mutant NSCLC tumors with varying co-mutations and associations with TMB and PD-L1 may better inform the responsiveness of these tumors to ICIs. There have now been several meta-analyses as well as real-world retrospective studies to determine the predictive impact of *KRAS* mutations in advanced or metastatic NSCLC patients (46, 47). Most studies did not demonstrate any differences in survival based on *KRAS* mutational status when patients received ICIs alone or in combination with chemotherapy. In contrast, a large meta-analysis including 386 *KRAS*-mutant and 927 *KRAS* wild-type NSCLC patients saw significant improvements in overall and progression-free survival in *KRAS*-mutant NSCLC patients receiving ICIs in first or second-line with or without chemotherapy compared to chemotherapy alone and significantly longer overall survival in *KRAS*-mutant compared to *KRAS* wild-type NSCLC (48). There are far fewer investigations into the heterogeneous *KRAS* subtypes that include different *KRAS* mutations, additional co-mutations, and responsiveness to ICIs. *STK11* and *KEAP1* mutations are often co-occurring and enriched in *KRAS* mutant tumors as seen here (**Figure 3**; **Supplementary Table S5**) (49). In a study of 1,194 *KRAS*-mutant NSCLC patients comparing *KRAS* G12C versus *KRAS* non-G12C mutations, co-mutation patterns of *STK11* and *KEAP1* were similar, and no significant differences were observed in median overall survival to single-agent ICI (50). Additional studies are warranted to determine the effectiveness of ICIs with these unique *KRAS* mutant heterogeneous subtypes, particularly in *KRAS* G12C mutant tumors where the efficacy of ICI therapy in comparison to targeted therapies remains unknown.

#### 4.3.3 Strong associations between *BRAF* V600E and PD-L1 expression compared to non-*BRAF* V600E and high TMB

Upon review of guideline-recommended driver genes with FDA-approved therapies (**Figure 1B**), we observed that some of the highest percentage of PD-L1 TPS ≥50% tumors harbored *BRAF* V600E mutations (46%) (**Figure 2D**) compared to other driver gene variants (*KRAS, EGFR, ALK, ERBB2, ROS1*). In contrast, these tumors had very low median TMB (4.6 mut/Mb). These correlations between *BRAF* mutant tumors, high PD-L1 expression, and low TMB levels have been witnessed in other studies (16, 22, 51–54). When comparing by variant type, tumors with non-*BRAF* V600E mutations were among the highest median TMBs (10.9 mut/Mb), compared to other guideline-recommended gene variants, including *BRAF* V600E tumors (**Figure 2D**). Negrao et al. (22) examined response to ICIs given alone or in combination with chemotherapy in patients with tumors harboring *BRAF* V600E, *BRAF* non-V600E, or other driver alterations, and showed that *BRAF* V600E and non-*BRAF* V600E driven tumors had superior progression-free and overall survival compared to *EGFR, ALK, HER2, KRAS, MET, ROS1* or *RET* driven tumors. Results in other studies found favorable responses to ICI therapy in *BRAF* mutant tumors, but equivalent survival to *BRAF* wild-type or NSCLC patient tumors with no driver mutations; however, specific *BRAF* variant types were not always included, many only examined the effects of ICI monotherapy, and/or correlation with variant type and PD-L1/TMB biomarkers was not examined with these cross-comparisons. A recent retrospective study by Wang et al. (52) determined that *BRAF*-mutant NSCLC patients treated with ICI plus chemotherapy resulted in better outcomes than those treated with chemotherapy or targeted therapy alone, suggesting ICI/chemo combinations could be given as first-line therapy for *BRAF*-mutant patients. Given the high PD-L1 expression found in *BRAF* V600E mutant tumors and the very high TMB levels in non-*BRAF* V600E mutant tumors, ICI/chemotherapy may be a highly effective choice as first-line therapy, with the option to provide approved targeted therapies for *BRAF* V600E NSCLC patients after progression.

#### 4.3.4 Genomic heterogeneity among tumors with *MET* amplified versus *MET* exon 14 skipping mutations

It is believed that genomic heterogeneity with regards to co-mutations and associations with ICI predictive biomarkers is vastly different among tumors with *MET* amplifications versus *MET* exon 14 skipping mutations, leading to differences in response to ICIs. Here, we show *MET* exon 14 skipping mutations frequently have high PD-L1 expression (TPS ≥50%), but low median TMB (5.5 mut/Mb) (**Figure 2C**). In comparison, tumors with *MET* amplifications have the highest frequency of PD-L1 TPS ≥50% expressing tumors out of all the driver mutations tested (70%) and a high median TMB (10.2 mut/Mb) (**Figure 2C**). Findings from other studies of *MET-*amplified tumors show varying correlations with PD-L1 and TMB depending on the method used to detect *MET* amplification (55). Though some studies of NSCLC patients with *MET* alterations have shown modest response to ICI therapy, a recent analysis found *MET* amplified tumors, but not those with *MET* exon 14 skipping mutations, had significantly improved overall survival compared to chemotherapy when given ICI after progression on first-line chemotherapy (56). Though there are no approved targeted therapies for NSCLC patients with *MET* amplifications, this alteration has been recognized as an emerging biomarker due to evidence of clinical activity against *MET*-amplified tumors with TKIs approved for *MET* exon 14 skipping mutations (13). For *MET*-amplified tumors with very high PD-L1 (TPS ≥50%) and high TMB (>10 mut/Mb), ICIs could be an effective treatment strategy for first-line therapy. However, co-mutations and inhibition of key immune signaling genes by *MET* alterations that impact tumor immunogenicity must be considered as they can impact response to ICIs (57, 58). In addition, studies with combined *MET* and *EGFR* TKIs to overcome acquired *MET* amplification in tumors resistant to *EGFR* TKIs have shown varying levels of response and toxicity profiles (56). ICI therapy in combination with *EGFR* TKIs in tumors with acquired *MET*-amplification may be an interesting alternative to combat resistance.

#### 4.3.5 Less frequent driver gene alterations (*ERBB2*, *ROS1*) and association with biomarkers of ICI response

There are far fewer investigations into the associations between *ERBB2* and *ROS1* driver genes with ICI biomarkers, co-mutations, and their influence on ICI response. One recent study assessing *ROS1* fusions using whole-transcriptome (59) suggested a large percentage of tumors harboring *ROS1* fusions are high expressers of PD-L1 (TPS ≥50%), while another study utilizing targeted NGS assays (60) suggested a frequency (35%) closer to what we observed in our study (29%; **Figure 2E**). *ERBB2*-altered NSCLC tumors with high PD-L1 expression are infrequent and typically have low TMB (10, 16), but evidence suggests the clinical characteristics, genomic landscape of co-mutations, response to TKIs, and associations with ICI predictive biomarkers are distinctly different among NSCLC tumors with *ERBB2* mutations versus amplifications (61–63). Here, *ERBB2* amplified tumors had one of the highest median TMBs (10.9 mut/Mb) compared to all other guideline-recommended oncogenic driver genes and *ERBB2* Y772-A775dup (5 mut/Mb) (**Figure 2F**), the most frequent *ERBB2* mutation found in NSCLC tumors. *ERBB2* amplified NSCLC tumors with high TMB were corroborated in a small study of metastatic NSCLC patients, however, minimal response to ICI therapy was observed (64). The limited data available suggests modest to poor responses for patients with NSCLC *ERBB2* and *ROS1*-driven tumors receiving ICI monotherapy or combination therapy, regardless of PD-L1 expression or TMB status, indicating additional co-mutations and other factors may play a role.

### 4.4 Importance of identifying oncogenic drivers before first-line ICI therapy

Broad coverage CGP has become increasingly important for patients with advanced NSCLC tumors with targetable oncogenic drivers considering evidence supporting lack of efficacy and even harm to NSCLC patients receiving ICI. Response to ICI or ICI plus chemotherapy is often limited or still under investigation in the presence of a therapy-associated oncogenic driver, regardless of PD-L1 expression or TMB status (8). CGP is even more important in advanced NSCLC patients who are non-smokers, as they are more likely to have tumors with targetable driver alterations.

ICI, with or without chemotherapy, is now routinely used as first-line therapy in advanced NSCLC patients with PD-L1 tumor expression ≥1%. Our analysis reveals that most tumors with therapy-associated driver mutations have low/intermediate (1 - 49%) PD-L1 expression, with 8% to 70% having high (≥50%) PD-L1 expression depending on the underlying driver mutations (**Figure 2**; **Supplementary Figure S1**). If CGP is not ordered, or test results have not been received before first-line treatment decision making, there is an increased risk of advanced NSCLC patients with tumors containing driver mutations receiving ICI therapy that would be ineffective or potentially dangerous. This is particularly concerning in advanced NSCLC patients with *EGFR* tumor mutations receiving sequential ICI therapy followed by osimertinib, as they experienced severe immune-related adverse events requiring hospitalization (65). Timing of osimertinib or ICI therapy for those with *EGFR* exon 19 deletions or exon 21 L858R mutations and high (≥50%) PD-L1 expression is complex, as these patients have worse progression-free and overall survival when receiving osimertinib as first-line therapy (66). Further studies are indicated in this cohort of patients to determine if the combination of osimertinib plus chemotherapy can combat potential resistance. Finally, a small subset of NSCLC patients can experience rapid accelerations of tumor growth or hyper-progressive disease upon receipt of ICI therapy (67). Though evidence of predictive biomarkers for this phenomenon is limited, some studies suggest co-occurrence between *MDM2* amplification or *EGFR* mutations, which we also saw evidence of (**Figure 3**; **Supplementary Table S5**), could be contributing to the progression.

Additionally, as shown by our final analysis, more complex relationships exist between the increasing number of tightly co-occurring alterations and increasing or decreasing levels of PD-L1 and TMB (**Figure 4**), which may have certain implications for guiding clinical decisions regarding ICI treatment. Although some of the TMB changes were found to be subtle (e.g., **Figures 4F, H**), the addition of one (**Figure 4H**) or two (**Figure 4F**) variants in these gene modules seems to push TMB levels from below 10 (i.e., low TMB) to ≥10 (i.e., high TMB). This subtle change may or may not on its own have a significant influence on the immunogenicity of the tumor itself. Still, it places patients in the high TMB category, making them eligible for certain ICI treatments (68). However, TMB levels do not tell the full story and a better clinical guide for ICI response involves looking at changes in TMB and PD-L1 together, which was a goal of this analysis. For the *KRAS–*associated gene module (**Figure 4F**), we detected a subtle change in increasing TMB in conjunction with a 10-12% increase in high PD-L1 IHC cases, which together may signify that patients with more variants in this gene module might benefit from ICI treatment. Alternatively, for the *STK11/KEAP1–*associated gene module (**Figure 4H**), although we see subtle changes in increasing TMB, we see the opposite trend for PD-L1 with a 15-22% reduction in high PD-L1 cases, suggesting that more variants in this gene cluster might influence TMB levels, but may potentially be reducing the immunogenicity of the tumor, making the patient less likely to benefit from ICI treatment. These are just a couple of examples of the complex relationships we observed between tightly co-occurring alterations and increasing or decreasing levels of PD-L1 and TMB, and further investigation is warranted to tease apart the mechanisms of these interactions.

## 5 Conclusion

In this study, we retrospectively analyzed real-world CGP data from 7,606 advanced and metastatic NSCLC tumors > using the broad coverage TSO 500 assay. We revealed heterogeneity in tumor genomic alterations and associations with biomarkers of ICI response. Co-occurrence analysis followed by network analysis with gene module detection revealed the presence of tightly co-occurring genomic alterations and allowed further characterization of their relationships with PD-L1 and TMB. Altogether, this data provides further characterization of NSCLC at the genomic and ICI response biomarker level and shows the clinical utility of broad coverage CGP testing, as performed by the TSO 500, in detecting both known and novel facets of NSCLC to inform treatment decision-making. Additionally, it produced evidence for future studies to determine if the findings presented here influence actual clinical response data to ICIs and molecular monotherapy agents.

## Data Availability

The data and code presented in the study are deposited in Zenodo, accession number 13137232 (https://zenodo.org/record/13137232). Raw sequencing data were derived from routine clinical testing of real-world patients and cannot be shared publicly. Further data inquiries can be directed to the corresponding author.
