# Supplementary material for: Genomic profiling of NSCLC tumors with the TruSight oncology 500 assay provides broad coverage of clinically actionable genomic alterations and detection of known and novel associations between genomic alterations, TMB, and PD-L1
Source: Front Oncol. 2024 Nov 27;14:1473327. doi: 10.3389/fonc.2024.1473327 (PMC11631745; doi:10.3389/fonc.2024.1473327)
Supplement: Supplementary file 1 [file DataSheet1.pdf]

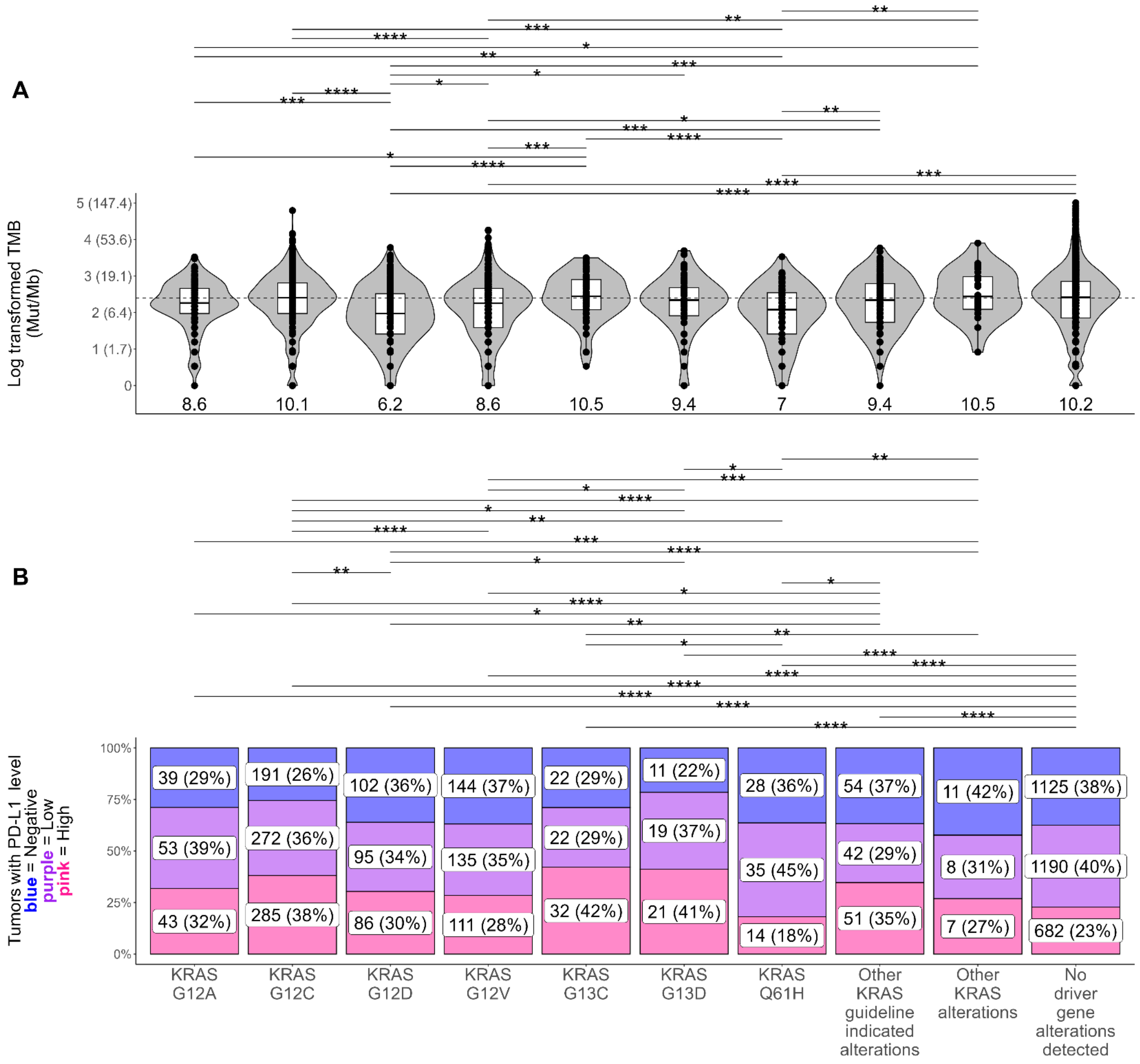

Supplemental Figure 1. Associations between the presence of known KRAS driver mutations, TMB, and PD-L1 in NSCLC tumors.

Differences in **(A)** tumor mutational burden (TMB; in mutations/Mb) and **(B)** PD-L1 tumor proportion score (TPS) were tested between NSCLC tumors with known driver/non-driver mutations within *KRAS*. For plots of TMB **(A)**, each dot in the box-violin plots represents an individual tumor from a unique patient falling into a particular variant group (X-axis) and is plotted along the Y-axis based on its log-transformed TMB value (non-transformed TMB value of each transformed value shown in parentheses on the Y-axis for clarity). The bottom, middle, and top horizontal boundaries of each box in the box-violin plots represent the first, second (median), and third quartiles of the data. The lines extending from the two ends of each box represent 1.5x outside the interquartile. Points

beyond the lines are considered outliers. The width of the grey-shaded regions around the boxes represents the density of the data points, whereas wider areas correspond to higher data point density. For plots of PD-L1 TPS level **(B)**, each section of the bar plots represents the percent of tumors falling in a particular variant group (X-axis) that were deemed to be negative (<1%), low (1-49%), or high (≥50%) for PD-L1 TPS. Differences in TMB and PD-L1 between groups were tested using linear regression and penalized likelihood ratio tests, respectively, adjusted for NSCLC histology. Results of testing can be found in **Supplementary Table 4**. *, P<0.05; **, P<0.01; ***, P<0.001; ****, P<0.0001.

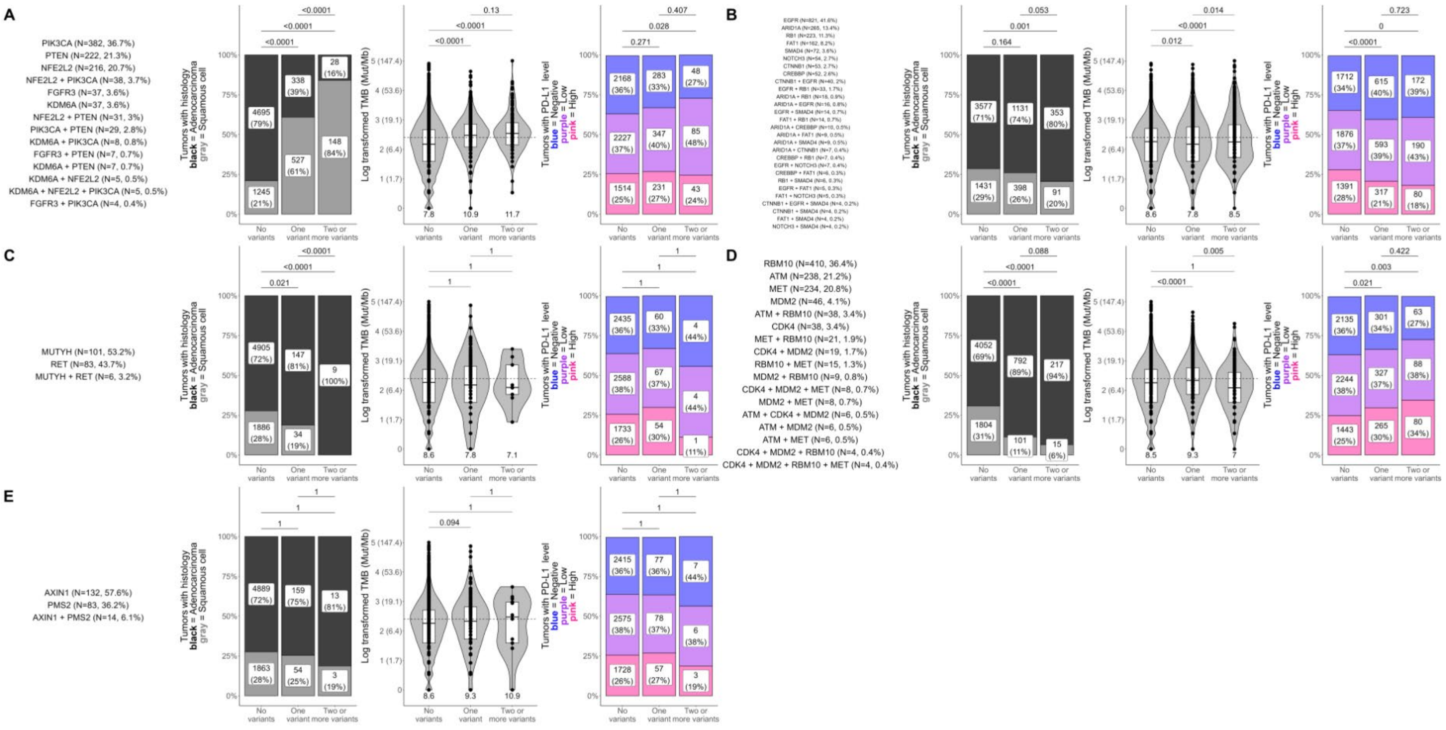

Supplemental Figure 2. Dose-dependent influences of gene module alteration number on histology, TMB, and PD-L1 for *PIK3CA*, *EGFR*, *MUTYH/RET*, *RBM10/MET*, and *AXIN1/PMS2*-associated gene modules.

Differences in histology, tumor mutational burden (TMB; in mutations/Mb), and PD-L1 tumor proportion score (TPS) were tested between NSCLC tumors with none, one, or ≥2 genomic alterations within genes co-occurring in each network-derived gene module. Analyses were performed for all gene modules and results for *DNMT3A*/*TET2*, *EGFR*, *RBM10*/*MET*, *AXIN1*, and *KRAS*-associated gene modules are shown here. The first column for each gene module shows the unique combinations of genes that had co-occurring alterations in the gene module. For clarity, only gene combinations detected in at least 4 patient tumors are shown. The remaining plots show differences in the distribution of histology (adenocarcinoma vs squamous cell carcinoma), TMB, and PD-L1 between tumors that had none, one, or ≥2 genomic alterations in genes of the gene module. For plots of TMB **(third column)**, each dot in the box-violin plots represents an individual tumor from a unique patient falling into a particular variant group (X-axis) and is plotted along the Y-axis based on its log-transformed TMB value (non-transformed TMB value of each transformed value shown in parentheses on the Y-axis for clarity). The

bottom, middle, and top horizontal boundaries of each box in the box-violin plots represent the first, second (median), and third quartiles of the data. The lines extending from the two ends of each box represent 1.5x outside the interquartile. Points beyond the lines are considered outliers. The width of the grey-shaded regions around the boxes represents the density of the data points, whereas wider areas correspond to higher data point density. Values below each box-violin plot is the untransformed median TMB of the group. For plots of PD-L1 TPS level **(last column)**, each section of the bar plots represents the percent of tumors falling in a particular variant group (X-axis) that were deemed to be negative (<1%), low (1-49%), or high (≥50%) for PD-L1 TPS. Differences in histology, TMB, and PD-L1 were tested using Fisher's exact test, t-test, and chi-squared test, respectively.
